# Cognitive Behavior Therapy With and Without Narrative Assessment and Suicide Attempts: A Systematic Review and Meta-Analysis

**DOI:** 10.1001/jamanetworkopen.2025.44632

**Published:** 2025-11-20

**Authors:** Wilco C. Janssen, Saskia Y. M. Mérelle, Wouter van Ballegooijen, Renske Gilissen, Claudi L. H. Bockting

**Affiliations:** 1 113 Suicide Prevention, Amsterdam, the Netherlands; 2 Department of Psychiatry, Amsterdam University Medical Centre, Amsterdam, the Netherlands; 3 Department of Clinical, Neuro and Developmental Psychology, Vrije Universiteit Amsterdam, Amsterdam, the Netherlands; 4 Centre for Urban Mental Health, Institute for Advanced Study, University of Amsterdam, Amsterdam, the Netherlands

## Abstract

**Importance:**

There is currently no evidence-based method to identify the forces that may drive someone to attempt suicide.

**Objective:**

To examine whether cognitive behavior therapy (CBT) interventions that incorporate a narrative assessment are associated with a greater reduction in suicide attempts than comparable interventions without this component.

**Data Sources:**

Studies were identified through version 25.0.1 of the Metapsy Suicide Prevention Database, which includes randomized clinical trials published up to April 2025 retrieved through PubMed, Embase, Web of Science, Scopus, and Cochrane Central, including unpublished studies and references from relevant articles.

**Study Selection:**

Included studies were randomized clinical trials of CBT interventions reporting suicide attempts as an outcome. Studies using waiting list controls, reporting only suicidal ideation, or lacking information on the type of assessment used were excluded.

**Data Extraction and Synthesis:**

Two reviewers independently extracted data and assessed risk of bias using the Cochrane Risk of Bias 2 tool. Meta-analyses were conducted using 3-level models with robust variance estimation. Relative risks (RRs) were pooled using the Mantel-Haenszel method. Analyses followed Preferred Reporting Items for Systematic Reviews and Meta-Analyses reporting guidelines. Data were pooled using a random-effects model.

**Main Outcomes and Measures:**

The primary outcome was the incidence of suicide attempts. Interventions were grouped by presence or absence of a narrative assessment component.

**Results:**

Twenty-three studies with 3262 participants met inclusion criteria. CBT interventions including a narrative assessment were associated with a significantly reduced risk of suicide attempt compared with controls (RR, 0.68; 95% CI, 0.53-0.87; 1764 participants across 14 studies), whereas CBT interventions without this component were not associated with risk of suicide attempt (RR, 1.17; 95% CI, 0.63-2.20; 1498 participants across 9 studies). Subgroup comparison indicated a significant difference between groups (*Q_1_ *= 7.27; *P* = .007; *I^2^* = 86%). Studies without a narrative assessment had significantly younger participants, a lower event rate, and slightly higher risk of bias.

**Conclusions and Relevance:**

In this systematic review and meta-analysis, CBT interventions including a narrative assessment were associated with a reduced risk of suicide attempt, while CBT without this component did not have an association with risk of suicide attempt. Age differences between study populations may partly explain this finding, as interventions in younger populations often show smaller and more inconsistent effect sizes. These results suggest that a narrative assessment may be a simple and effective way to capture the forces that lead to suicide attempts and to direct interventions toward their prevention. Causal conclusions require direct head-to-head trials.

## Key Points

**Question:**

Are cognitive behavioral interventions that include a narrative assessment associated with a greater reduction in the risk of suicide attempts than those without?

**Findings:**

In this meta-analysis of 23 randomized clinical trials with 3262 participants, interventions incorporating a narrative assessment were associated with reduced suicide attempts, while those without were not.

**Meaning:**

These findings suggest that including a narrative assessment may enhance the effectiveness of interventions in preventing suicide attempts.

## Teaser

This systematic review and meta-analysis of randomized clinical trials examines the association between cognitive behavioral interventions with vs without a narrative assessment and risk of suicide attempt.

## Introduction

To reduce the risk of a suicide attempt, practitioners are often tasked with the challenge to identify the forces that might drive individuals to attempt suicide before they actually do. The only available method to aid clinicians in this task is the Collaborative Assessment and Management of Suicidality (CAMS^1^) framework, which has been specifically designed to identify suicidal drivers. Meta-analysis shows CAMS has a positive association with suicidal thoughts, hopelessness, and the therapeutic alliance, but does not reduce suicide attempts compared with controls.^2^ Although reducing suicidal thoughts and other risk factors for suicide is valuable, it remains uncertain whether this translates into the prevention of suicide attempts: ideation-to-action theories posit that both are influenced by different factors^3^ and interventions often decrease one or the other, not both.^2,4,5,6^ It is therefore important to develop new ways to identify risk and protective factors for suicide attempts and gear treatments toward their prevention.

Insights may be derived from the way in which interventions with proven efficacy in preventing suicide attempts select treatment targets. Brief cognitive behavior therapy for suicide prevention (BCBT)^6^ uses a procedure called a narrative assessment to this end, which also occurs in dialectical behavior therapy under the name chain analysis.^7^ During a narrative assessment, practitioner and patient first identify the specific moment in the past weeks in which the patient came closest to suicide (referred to as the index crisis or worst point). For someone who recently made a suicide attempt, the index crisis is this suicide attempt, for someone who has not made a suicide attempt, the index crisis is the moment at which they came closest to attempting suicide. Once the index crisis has been identified, the practitioner asks the patient to tell the story of this situation and practitioner and patient work together to gain a deeper understanding of the sequence of events, thoughts, emotions, and behaviors that lead up to this peak, including factors that helped delay or reduce it. The practitioner is careful to prevent the conversation from drifting toward issues that were not present during the index crisis.

This seems like a sensible approach to catch the factors in action that may one day drive a patient to make a (or another) suicide attempt. Preliminary support for the use of a narrative assessment comes from a study^8^ in which all participants were offered the same intervention, preceded by either a narrative assessment or a structured interview. Participants whose treatment began with the narrative assessment experienced fewer suicidal thoughts after their treatment than participants whose treatment started with the structured interview. However, the study did not examine the impact on suicide attempts. This study therefore aims to conduct a systematic review and meta-analysis, in which we investigate the association of comparable interventions with and without a narrative assessment with the incidence of suicide attempts.

## Methods

This systematic review and meta-analysis was conducted and reported in accordance with the Preferred Reporting Items for Systematic Reviews and Meta-Analyses (PRISMA) reporting guideline and was preregistered in PROSPERO (CRD42024604281). This systematic review and meta-analysis used the Metapsy.org Suicide Prevention database,^9^ a database of randomized clinical trials on psychological interventions, that is regularly updated on the basis of rigorous systematic reviews. This study used version 25.0.1, which will be released later this year and spans all trials up to April 2025, retrieved via PubMed, Embase, Web of science, Scopus, and the Cochrane Central Register of Controlled Trials. Unpublished studies and studies retrieved from the reference lists of relevant articles were also included. To construct and update the Metapsy database, 2 researchers independently searched and selected studies, extracted data from the trials, and assessed risk of bias. Any disagreements were resolved by discussion and, if necessary, in consultation with a third researcher. Decisions were recorded in Covidence. The full search string can be found in Supplement 1.

### Study Selection

Studies were first screened for eligibility on titles and abstracts and then on full text. Studies were included in the MetaPsy database if they:

- Had a randomized design comparing 2 or more groups;
- Reported suicide attempts, suicide, self-harm leading to hospitalization, or suicidal thoughts as an outcome (self-harm was only included if the study’s definition of self-harm included suicide attempts);
- Were written in English, Dutch, German, or Greek; and
- Studied a psychological intervention containing at least 1 psychotherapeutic technique.

For the purpose this systematic review and meta-analysis, studies were excluded if:

- They only reported suicidal ideation, not suicide attempts;
- They studied psychological interventions other than cognitive behavioral therapy (CBT), since we wanted to limit variation between the groups, other than the presence or absence of a narrative assessment, and we expected there would be more studies on CBT than on other interventions;^10^
- They used a waiting list control, since this can lead to overestimation of effect sizes; or
- It could not be established whether the intervention included a narrative assessment.

### Data Extraction

The first author, year of publication, number of randomized participants, mean age of participants, percentage of females, setting, recruitment strategy, main *Diagnostic and Statistical Manual of Mental Disorders* (Fifth Edition) classification of participants, inclusion and exclusion criteria, type of psychotherapy, type of control condition, the number of suicide attempts, and effect size were recorded for each trial. In case any of these characteristics could not be extracted from the article, the authors were contacted twice via email before the data were considered unavailable. To limit the amount of variation between the interventions, other than the presence or absence of a narrative interview, trials of third-wave therapies and dialectical behavior therapy were excluded, although they are sometimes considered forms of CBT. For trials on CBT, researchers also recorded whether a narrative assessment was or was not included. If this information could not be extracted from the article, the corresponding and last author were each contacted via email once. Study characteristics were tabulated to assess their eligibility for each synthesis.

### Risk of Bias Assessment and Data Preparation

During the construction and update of the Metapsy database, all studies were assessed for risk of bias by 2 researchers using version 2 of the Cochrane Collaboration Risk of Bias Tool for randomized trials, which assesses possible sources of bias due to the randomization process, deviations from intended interventions, missing outcome data, measurement of the outcome, and selection of the reported results.Relative risk was calculated for each trial as an effect size.

### Statistical Analysis

Mean age, sample size, number of sessions, study duration in weeks, and risk of bias were calculated for studies with and without a narrative assessment and compared using the Mann-Whitney *U* test. Three separate meta-analysis were conducted with the metapsyTools package in R (version 4.4.1) and R studio (version 2024.04.2 + 764) (R Project for Statistical Computing) and summarized in forest plots. First, the effect sizes of interventions with and without a narrative assessment were pooled in 2 separate meta-analyses. Both studies with and without a narrative assessment were then included in a third meta-analysis to directly compare their effect sizes in subgroup analysis.

Three-level models were estimated with effect sizes nested in studies to account for multiarm trials, applying robust variance estimation. We assumed an intrastudy correlation of 0.6. Relative risk ratios (RR) were pooled using the Mantel-Haenszel method to account for zero counts.. An RR of 0.90 to 0.70 was considered small; an RR of 0.69 to 0.50, medium; and an RR smaller than 0.50, large.^11^ Statistical significance was determined with a 2-sided *P *value of <.05

Prediction intervals, τ, the *I*^2^ statistic, and their 95% CIs were calculated as indicators of heterogeneity, with *I*^2^ values of 0%, 25%, 50%, and 75% considered to indicate no, low, moderate, and high heterogeneity, respectively.^12^ Outlier and influence analyses were performed if *I*^2^ was equal to or higher than 50%. The robustness of the findings was tested by (1) excluding outliers with a CI that did not overlap with that of the pooled effect size; (2) excluding influential cases according to the methods by Viechtbauer and Cheung^13^; (3) adjusting for publication bias with the limit meta-analysis method^14^; and (4) repeating the meta-analysis with studies with a low risk of bias.

Sensitivity analysis was performed to assess the influence of 4 factors, but only when at least 4 studies remained after accounting for the respective factor: (1) the exclusion of people with severe suicidal thoughts and behaviors from the trial,which might cause range restriction and other methodological issues; (2) age younger than 25 years, since interventions for young people tend to find smaller and more inconsistent results than interventions for adults^15^; (3) online interventions, since evidence that online interventions can be effective in preventing suicide attempts is sparse^16,17^; and (4) interventions with an above-average treatment length, since a quick treatment effect is of great importance in suicide prevention and longer treatments have been found to be somewhat more effective than shorter treatments.^10^ Differences in treatment length might therefore affect the outcomes of the meta-analysis.

## Results

### Study Selection

**Figure 1.**
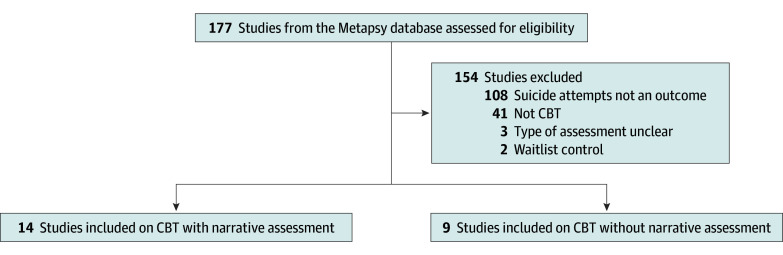
Study Selection Flowchart Abbreviations: CBT, cognitive behavioral therapy

Figure 1 shows a flowchart of the study selection with reasons for exclusion. A total of 23 randomized clinical trials, with 3262 participants, were included in the meta-analysis. Of these, 14 studies incorporated a narrative assessment,^6,17,18,19,20,21,22,23,24,25,26,27,28,29^ and 9 studies did not include a narrative assessment.^30,31,32,33,34,35,36,37^

### Descriptive Statistics

**Figure 2.**
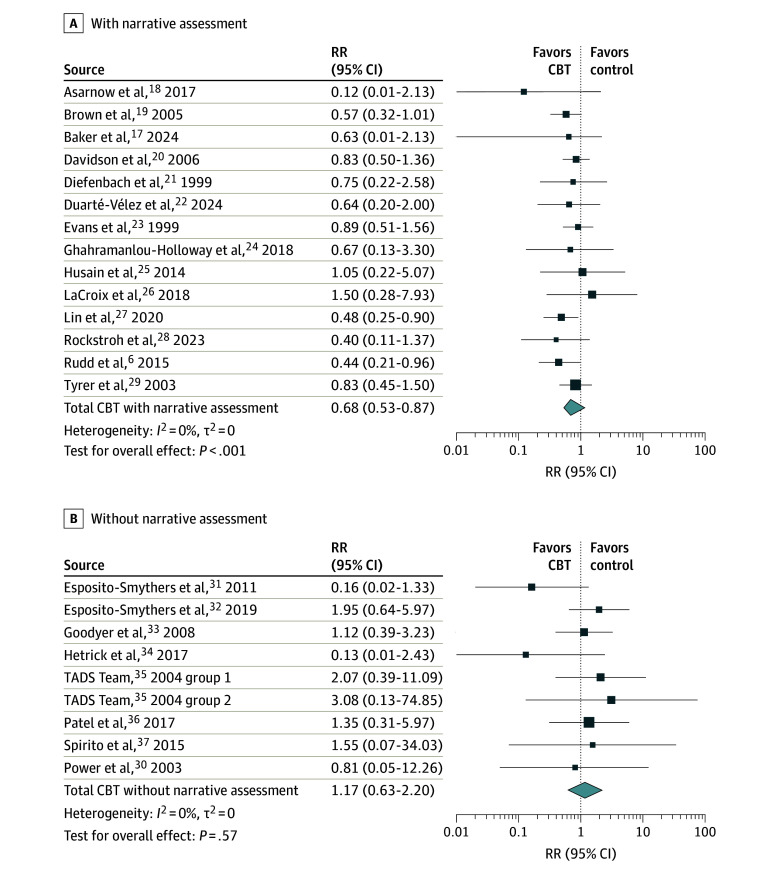
Association of CBT Interventions With and Without a Narrative Assessment With Risk of Suicide Attempt Abbreviations: RR, relative risk, TADS, Treatment for Adolescents With Depression Study

Descriptive statistics and comparisons between studies with and without a narrative assessment are shown in the Table and Figure 2. The group of studies that incorporated a narrative assessment included 14 randomized clinical trials^6,17,18,19,20,21,22,23,24,25,26,27,28,29^with a total of 1764 participants. The group without a narrative assessment comprised 9 trials^30,31,32,33,34,35,36,37^ with a total of 1498 participants.

**Table.** Descriptive Statistics and Statistical Comparisons of Studies With and Without a Narrative Assessment^a^

Variable	Narrative, mean (SD)		*P* value
	With	Without	
Studies, No.	14	9	NA
Participants, No.	1764	1498	NA
Suicide attempts, No.	258	52	NA
Participant age, y	27.08 (7.49)	18.18 (9.82)	.03
Sample size, No.	58.86 (53.05)	82.89 (68.44)	.36
Event rate	0.16 (0.14)	0.05 (0.06)	.01
Power	0.24 (0.23)	0.20 (0.17)	.68
Study duration, wk	27.38 (17.00)	23.44 (17.69)	.79
Treatment duration, sessions	8.83 (3.96)	12.87 (7.79)	.31
Intervention type			
Add-on, %	46.67	37.50	NA
Standalone, %	53.33	62.50	NA
Recruitment setting			
Inpatient, %	57, 14	22, 22	NA
Partial hospitalization, %	14, 29	11, 11	
Outpatient, %	35, 71	44, 44	
Emergency department, %	50, 00	00, 00	
Primary care, %	00, 00	11,11	
School, %	00, 00	11,11	
Risk of bias			
Randomization	1.29 (0.47); low	1.22 (0.44); low	.77
Deviations	1.36 (0.50); low	1.56 (0.53); low	.38
Missing data	1.64 (0.93); low	2.11 (1.05); some concern	.30
Measurement	1.50 (0.76); low	1.38 (0.52); low	.90
Reporting	1.79 (0.70); low	1.78 (0.67); low	.99
Overall	2.36 (0.50); some concern	2.89 (0.33); some concern	.02

Abbreviations: NA, not applicable.

^a^ Studies with a narrative assessment: Asarnow et al,^18^ 2017; Brown et al,^19^ 2005; Baker et al,^17^ 2024; Davidson et al,^20^ 2006; Diefenbach et al,^21^ 2024; Duarte-Velez et al,^22^ 2023; Evans et al,^23^ 1999; Ghahramanlou-Holloway et al,^24^ 2018; Husain et al,^25^ 2014; LaCroix et al,^26^ 2018; Lin et al,^27^ 2020; Rockstroh et al,^28^ 2023; Rudd et al,^6^ 2015; Tyrer et al,^29^ 2003. Studies without a narrative assessment: Esposito-Smythers et al,^31^ 2011; Esposito-Smythers et al,^32^ 2019; Goodyer et al,^33^ 2008; Hetrick et al,^34^ 2017; March et al,^35^ 2004; Patel et al,^36^ 2017; Power et al,^30^ 2003; Spirito et al,^37^ 2015.

The mean age of participants in the studies without a narrative assessment was significantly lower than that of participants in the studies with a narrative assessment (mean [SD] age, 18.18 [9.82] years vs 27.08 [7.49] years; *P* = .02). The studies without a narrative assessment, vs those with a narrative assessment, also showed a slightly higher overall risk of bias (mean [SD] with some concerns, 2.89 [0.33] vs 2.36 [0.05]; *P* = .01) and had a lower event rate (mean [SD], 0.05 [0.06] vs 0.16 [0.14]; *P* = .01). Most studies on CBT with a narrative assessment recruited participants through inpatient facilities and emergency departments, whereas studies on CBT without a narrative assessments predominantly recruited participants through outpatient facilities, schools, and in primary care. Other variables, including sample size, study duration, and treatment length, did not differ significantly between the groups.

### Meta-Analyses

#### Separate Meta-Analysis of CBT With a Narrative Assessment

CBT with a narrative assessment was associated with a significantly lower risk of suicide attempt compared with controls (RR, 0.68; 95% CI, 0.56-0.81; *P* < .001), with low heterogeneity between the 14 studies (1764 participants; τ = 0.00; 95% CI, 0.00-0.78; *I*^2^ = 0.00%; 95% CI, 0.00%-55.03%). The results of the meta-analysis remained unchanged when sensitivity analyses were conducted to account for outliers, influential cases, risk of bias, publication bias, age, the exclusion of people with severe suicidality, and online interventions. When excluding all studies with above-average treatment length, only 4 studies, with 207 participants, remained,^23,24,25,27^ and the finding was no longer statistically significant (RR, 0.69; 95% CI, 0.39-1.22; *P* = .13).

#### Separate Meta-Analysis of CBT Without a Narrative Assessment

CBT without a narrative assessment was not associated with the risk of suicide attempts (RR, 1.17; 95% CI, 0.63-2.20; *P* = .57). Again, heterogeneity between the 9 studies (with 1498 participants) was low (τ^2^ = 0; 95% CI, 0.00-1.71; *I*^2^ = 0.00%; 95% CI, 0.00%-64.80%), and the results were not impacted by accounting for outliers, influential cases, publication bias, risk of bias, online studies, and treatment length. When the 3 studies^33,35^ that excluded people with severe suicidality were removed from the analysis, the RR dropped below 1, but the result remained nonsignificant. Since only 1 of the studies^36^ without a narrative assessment was conducted among adults, it was not possible to conduct a sensitivity analysis for age or age group.

#### Comparison of CBT With and Without a Narrative Assessment in a Single Meta-Analysis

Together, CBT interventions with and without a narrative assessment were associated with a lower risk of suicide attempt (RR, 0.74; 95% CI, 0.58-0.93; *P* = .02). When subgroup analysis was conducted to compare CBT with and without a narrative assessment, a significant difference was found in favor of CBT with a narrative assessment (Q_1_ = 7.27, *P* = .007; *I^2^* = 86%).

## Discussion

The findings of this systematic review and meta-analysis indicate that CBT interventions incorporating a narrative assessment were associated with reduced risk of suicide attempts, with a medium effect size, suggesting that they are more impactful than CBT interventions without this component, which did not show a statistically significant result. These findings were robust across multiple sensitivity analyses.

The association between CBT with a narrative assessment and risk of suicide attempt became insignificant only when studies with an above average treatment length were excluded. However, this sensitivity analysis was based on just 4 studies, most of which had small sample sizes, and the findings should therefore be interpreted cautiously. Treatment length did not differ significantly between studies with and without a narrative component, suggesting that treatment duration alone cannot explain the difference in results. At the same time, the narrative component serves to identify rather than address treatment targets, and its impact is likely contingent on the intervention that follows. It may therefore be more impactful when sufficient treatment length is available to work through these identified targets.

To provide a more definitive answer to the question whether a narrative assessment can indeed improve the effectiveness of interventions, head-to-head comparisons are needed in which the impacts of identical interventions with and without a narrative assessment on suicide or suicide attempts (not suicidal ideation) are directly compared in randomized clinical trials. It may also be valuable to examine factors that influence the effectiveness of a narrative assessment. While a window of 4 weeks or less since a suicide attempt appears both workable and clinically meaningful,^39^ it remains uncertain whether a longer interval would still yield useful insights. This may differ between suicidal ideation and suicidal behavior: an instance of behavior may be easier to recall and reconstruct than an instance of thoughts or feelings. A related question is whether a narrative assessment based on an index episode of relatively mild ideation can adequately capture the processes that may eventually culminate in a suicide attempt, or whether such dynamics become visible only during episodes of more severe suicidal thoughts and behaviors. Finally, it seems plausible that a narrative assessment is more impactful when followed by a personalized treatment that can be tailored to the personal dynamics revealed through the interview, such as BCBT, rather than when embedded in a standardized, one-size-fits-all approach.

### Limitations

This study has limitations. It is important to realize that none of the studies in this meta-analysis were designed to isolate the effects of a narrative assessment. Although the included studies were all randomized clinical trials, any associations found in this meta-analysis are therefore associational, not necessarily causal, and can be influenced by several confounders.

Three such confounders were identified, one of which was a difference in the age of participants in studies with and without a narrative assessment: almost all studies on CBT with a narrative assessment were conducted among adults, while most studies on CBT without a narrative assessment focused on adolescents and young adults. This represents a major limitation of the literature, as studies involving younger populations often report smaller and more inconsistent effects,^15^ perhaps because most psychotherapies are directed at the individual, whereas the difficulties of young people are more often contextually and relationally driven.^38^ Consequently, the difference in participant age may have contributed to the larger uncertainty surrounding the outcomes associated with CBT without a narrative assessment and potentially led to an underestimation of the effect size of CBT without a narrative assessment. This may in turn be due to studies involving young people employing more stringent control conditions or safety protocols. However, the level of detail in the majority of studies does not permit this hypothesis to be adequately examined.

Apart from the difference in age, the event rate was significantly lower in studies without a narrative assessment, probably due to differences in the recruitment sites used. This limits the power to detect intervention effects in these trials. The overall risk of bias in studies without a narrative assessment was slightly higher but still fell within the same category of some concerns as the studies with a narrative assessment, suggesting that this difference is likely negligible.

## Conclusions

In this systematic review and meta-analysis of CBT interventions, CBT with a narrative assessment was associated with a reduced risk of suicide attempt, whereas CBT without this component was not. In light of this uncertainty, clinicians may do well to use CBT protocols that include a narrative assessment, such as the BCBT protocol from Bryan and Rudd.^40^

## Notes

Supplement 1. eAppendix. Search strings

Supplement 2. Data Sharing Statement
