# Supplementary material for: Cognitive Behavior Therapy With and Without Narrative Assessment and Suicide Attempts: A Systematic Review and Meta-Analysis
Source: JAMA Netw Open. 2025 Nov 20;8(11):e2544632. doi: 10.1001/jamanetworkopen.2025.44632 (PMC12635872; doi:10.1001/jamanetworkopen.2025.44632)
Supplement: Supplement 1. — eAppendix. Search strings [file jamanetwopen-e2544632-s001.pdf]

Supplemental Online Content

Janssen WC, Mérelle SM, van Ballegooijen W, Gilissen R, Bockting CH. CBT with and without narrative assessment and suicide attempts: a systematic review and meta-analysis. *JAMA Netw Open.* 2025;8(11):e2544632. doi:10.1001/jamanetworkopen.2025.44632

eAppendix. Search strings

This supplemental material has been provided by the authors to give readers additional information about their work.

© 2025 Janssen WC et al. *JAMA Network Open*.

eAppendix. Search strings

PubMed

Suicide

"Self-Injurious Behavior"[Mesh:NoExp] OR "suicide"[Mesh] OR “self-injur*”[tiab] OR “self-harm”[tiab] OR selfharm[tiab] OR suicid*[tiab] OR “self-kill*”[tiab] OR “self-poison*”[tiab] OR “self-stabb*”[tiab] OR parasuicide*[tiab]

Psychotherapy & online interventions

"Psychotherapy"[Mesh] OR “Counseling”[Mesh] OR "Self-Help Groups"[Mesh:NoExp] OR "Therapy, Computer-Assisted"[Mesh:NoExp] OR "Telemedicine"[Mesh] OR "Videoconferencing"[Mesh:NoExp] OR "Gamification"[Mesh] OR psychotherap*[tiab] OR aromatherap*[tiab] OR “art therap*”[tiab] OR “behavioral therap*”[tiab] OR “behavioral treatment*”[tiab] OR “behavioral intervention*”[tiab] OR “behavioural therap*”[tiab] OR “behavioural treatment*”[tiab] OR “behavioural intervention*”[tiab] OR “behavior therap*”[tiab] OR “behavior treatment*”[tiab] OR “behavior intervention*”[tiab] OR “behaviour therap*”[tiab] OR “behaviour treatment*”[tiab] OR “behaviour intervention*”[tiab] OR CBT[tiab] OR “cognitive psychotherap*”[tiab] OR “cognitive therap*”[tiab] OR bibliotherap*[tiab] OR “color therap*”[tiab] OR “crisis intervention*”[tiab] OR “dance therap*”[tiab] OR “emotion-focused therap*”[tiab] OR “psychological feedback”[tiab] OR “psychological treatment*”[tiab] OR “psychological intervention*”[tiab] OR “psychological therap*”[tiab] OR “sensory feedback”[tiab] OR “gestalt therap*”[tiab] OR hypnos*[tiab] OR imagery[tiab] OR “implosive therap*”[tiab] OR EMDR[tiab] OR “psychological desensitization*”[tiab] OR “desensitization reprocessing”[tiab] OR “desensitisation reprocessing”[tiab] OR “exposure therap*”[tiab] OR “music therap*”[tiab] OR grouptherap*[tiab] OR “group therap*”[tiab] OR “narrative therap*”[tiab] OR mindfulness[tiab] OR “play therap*”[tiab] OR “psychoanalytic therap*”[tiab] OR “reality therap*”[tiab] OR “socioenvironmental therap*”[tiab] OR “acceptance and commitment therap*”[tiab] OR “milieu therap*”[tiab] OR “problem-solving therap*”[tiab] OR “dialectical behavio*”[tiab] OR (DBT[tiab] AND dialectical*[tiab]) OR “interpersonal therap*”[tiab] OR (“future oriented”[tiab] AND training[tiab]) OR “collaborative assessment*”[tiab] OR CAMS[tiab] OR (systems[tiab] AND training[tiab] AND emotional[tiab] AND predictability[tiab] AND “problem solving*”[tiab]) OR STEPPS[tiab] OR (“mentalisation-based”[tiab] AND training[tiab]) OR “mentalisation-based therap*”[tiab] OR (“mentalization-based”[tiab] AND training[tiab]) OR “mentalization-based therap*”[tiab] OR counselling[tiab] OR counseling[tiab] OR “self-help group*”[tiab] OR “selfhelp group*”[tiab] OR “motivational interview*”[tiab] OR “blended face-to-face”[tiab] OR telehealth[tiab] OR “tele-health”[tiab] OR telepsychology[tiab] OR “tele-psychology”[tiab] OR telepsychiatry[tiab] OR “tele-psychiatry”[tiab] OR “tele-therap*”[tiab] OR teletherap*[tiab] OR “tele-medicine”[tiab] OR telemedicine[tiab] OR telecare[tiab] OR “tele-care”[tiab] OR teleconference*[tiab] OR “tele-conferenc*”[tiab] OR videoconferenc*[tiab] OR “video-conferenc*”[tiab] OR ehealth[tiab] OR “e-health”[tiab] OR “e-treat*”[tiab] OR “e-therap*”[tiab] OR mhealth[tiab] OR “m-health”[tiab] OR “internet-based*”[tiab] OR “internet treat*”[tiab] OR “internet intervention*”[tiab] OR “internet counsel*”[tiab] OR “distance counsel*”[tiab] OR “web-based*”[tiab] OR

© 2025 Janssen WC et al. *JAMA Network Open*.

cybercounsel*[tiab] OR “cyber-counsel*”[tiab] OR “online treat*”[tiab] OR “online therap*”[tiab] OR “online intervention*”[tiab] OR “online prevention*”[tiab] OR “online counsel*”[tiab] OR “e-counsel*”[tiab] OR ecounsel*[tiab] OR “serious game*”[tiab] OR “serious gaming”[tiab] OR gamification[tiab]

RCT, CT & other studies
(“Double-Blind Method”[Mesh] OR “Single-Blind Method”[Mesh] OR “Placebos”[Mesh] OR “Clinical trial”[pt] OR "Randomized Controlled Trial" [pt] OR “Random allocation” [Mesh] OR “Comparative study”[pt] OR “Evaluation studies”[pt] OR “cross-over studies”[Mesh] OR "clinical trial*"[tiab] OR "pragmatic trial*"[tiab] OR "real world trial*"[tiab] OR ((singl*[tiab] OR doubl*[tiab] OR trebl*[tiab] OR tripl*[tiab]) AND (mask*[tiab] OR blind*[tiab])) OR "latin square"[tiab] OR placebo*[tiab] OR random*[tiab] OR RCT[tiab] OR control[tiab] OR controll*[tiab]) NOT (“Animals”[Mesh] NOT “Humans”[Mesh])

Embase

Suicide
'suicidal behavior'/exp OR ‘self-injur*’:ab,ti,kw OR ‘self-harm’:ab,ti,kw OR selfharm:ab,ti,kw OR suicid*:ab,ti,kw OR ‘self-kill*’:ab,ti,kw OR ‘self-poison*’:ab,ti,kw OR ‘self-stabb*’:ab,ti,kw OR parasuicide*:ab,ti,kw

Psychotherapy & online interventions
'psychotherapy'/exp OR 'counseling'/exp OR 'self help'/exp OR 'computer assisted therapy'/de OR 'telemedicine'/exp OR 'videoconferencing'/exp OR 'gamification'/exp OR psychotherap*:ab,ti,kw OR aromatherap*:ab,ti,kw OR ‘art therap*’:ab,ti,kw OR ‘behavioral therap*’:ab,ti,kw OR ‘behavioral treatment*’:ab,ti,kw OR ‘behavioral intervention*’:ab,ti,kw OR ‘behavioural therap*’:ab,ti,kw OR ‘behavioural treatment*’:ab,ti,kw OR ‘behavioural intervention*’:ab,ti,kw OR ‘behavior therap*’:ab,ti,kw OR ‘behavior treatment*’:ab,ti,kw OR ‘behavior intervention*’:ab,ti,kw OR ‘behaviour therap*’:ab,ti,kw OR ‘behaviour treatment*’:ab,ti,kw OR ‘behaviour intervention*’:ab,ti,kw OR CBT:ab,ti,kw OR ‘cognitive psychotherap*’:ab,ti,kw OR ‘cognitive therap*’:ab,ti,kw OR bibliotherap*:ab,ti,kw OR ‘color therap*’:ab,ti,kw OR ‘crisis intervention*’:ab,ti,kw OR ‘dance therap*’:ab,ti,kw OR ‘emotion-focused therap*’:ab,ti,kw OR ‘psychological feedback’:ab,ti,kw OR ‘psychological treatment*’:ab,ti,kw OR ‘psychological intervention*’:ab,ti,kw OR ‘psychological therap*’:ab,ti,kw OR ‘sensory feedback’:ab,ti,kw OR ‘gestalt therap*’:ab,ti,kw OR hypnos*:ab,ti,kw OR imagery:ab,ti,kw OR ‘implosive therap*’:ab,ti,kw OR EMDR:ab,ti,kw OR ‘psychological desensitization*’:ab,ti,kw OR ‘desensitization reprocessing’:ab,ti,kw OR ‘desensitisation reprocessing’:ab,ti,kw OR ‘exposure therap*’:ab,ti,kw OR ‘music therap*’:ab,ti,kw OR grouptherap*:ab,ti,kw OR ‘group therap*’:ab,ti,kw OR ‘narrative therap*’:ab,ti,kw OR mindfulness:ab,ti,kw OR ‘play therap*’:ab,ti,kw OR ‘psychoanalytic therap*’:ab,ti,kw OR ‘reality therap*’:ab,ti,kw OR ‘socioenvironmental therap*’:ab,ti,kw OR ‘acceptance and commitment therap*’:ab,ti,kw OR ‘milieu therap*’:ab,ti,kw OR ‘problem-solving therap*’:ab,ti,kw OR ‘dialectical behavio*’:ab,ti,kw OR (DBT NEAR/3 dialectical*):ab,ti,kw OR ‘interpersonal

© 2025 Janssen WC et al. *JAMA Network Open*.

therap*':ab,ti,kw OR ('future oriented' NEAR/3 training):ab,ti,kw OR 'collaborative assessment*':ab,ti,kw OR CAMS:ab,ti,kw OR (systems NEAR/1 training NEAR/2 emotional NEAR/1 predictability NEAR/2 'problem solving*'):ab,ti,kw OR STEPPS:ab,ti,kw OR ('mentalisation-based' NEAR/3 training):ab,ti,kw OR 'mentalisation-based therap*':ab,ti,kw OR ('mentalization-based' NEAR/3 training):ab,ti,kw OR 'mentalization-based therap*':ab,ti,kw OR counselling:ab,ti,kw OR counseling:ab,ti,kw OR 'self-help group*':ab,ti,kw OR 'selfhelp group*':ab,ti,kw OR 'motivational interview*':ab,ti,kw OR 'blended face-to-face':ab,ti,kw OR telehealth:ab,ti,kw OR 'tele-health':ab,ti,kw OR telepsychology:ab,ti,kw OR 'tele-psychology':ab,ti,kw OR telepsychiatry:ab,ti,kw OR 'tele-psychiatry':ab,ti,kw OR 'tele-therap*':ab,ti,kw OR teletherap*:ab,ti,kw OR 'tele-medicine':ab,ti,kw OR telemedicine:ab,ti,kw OR telecare:ab,ti,kw OR 'tele-care':ab,ti,kw OR teleconference*:ab,ti,kw OR 'tele-conferenc*':ab,ti,kw OR videoconferenc*:ab,ti,kw OR 'video-conferenc*':ab,ti,kw OR ehealth:ab,ti,kw OR 'e-health':ab,ti,kw OR 'e-treat*':ab,ti,kw OR 'e-therap*':ab,ti,kw OR mhealth:ab,ti,kw OR 'm-health':ab,ti,kw OR 'internet-based*':ab,ti,kw OR 'internet treat*':ab,ti,kw OR 'internet intervention*':ab,ti,kw OR 'internet counsel*':ab,ti,kw OR 'distance counsel*':ab,ti,kw OR 'web-based*':ab,ti,kw OR cybercounsel*:ab,ti,kw OR 'cyber-counsel*':ab,ti,kw OR 'online treat*':ab,ti,kw OR 'online therap*':ab,ti,kw OR 'online intervention*':ab,ti,kw OR 'online prevention*':ab,ti,kw OR 'online counsel*':ab,ti,kw OR 'e-counsel*':ab,ti,kw OR ecounsel*:ab,ti,kw OR 'serious game*':ab,ti,kw OR 'serious gaming':ab,ti,kw OR gamification:ab,ti,kw

RCT, CT & other studies
'crossover procedure'/exp OR 'double blind procedure'/exp OR 'randomized controlled trial'/exp OR 'single blind procedure'/exp OR 'placebo'/exp OR 'clinical trial'/exp OR 'comparative study'/de OR 'evaluation study'/exp OR 'clinical trial*':ab,ti,kw OR 'pragmatic trial*':ab,ti,kw OR 'real world trial*':ab,ti,kw OR ((singl*:ab,ti,kw OR doubl*:ab,ti,kw OR trebl*:ab,ti,kw OR tripl*:ab,ti,kw) AND (mask*:ab,ti,kw OR blind*:ab,ti,kw)) OR 'latin square':ab,ti,kw OR placebo*:ab,ti,kw OR random*:ab,ti,kw OR RCT:ab,ti,kw OR control:ab,ti,kw OR controll*:ab,ti,kw

PsycINFO

Suicide
DE "Suicide" OR DE "Attempted Suicide" OR DE "Suicidality" OR DE "Suicidal Ideation" OR DE "Suicide Prevention" OR DE "Suicidology" OR DE "Self-Poisoning" OR TI("self-injur*" OR "self-harm" OR selfharm OR suicid* OR "self-kill*" OR "self-poison*" OR "self-stabb*" OR parasuicide*) OR AB("self-injur*" OR "self-harm" OR selfharm OR suicid* OR "self-kill*" OR "self-poison*" OR "self-stabb*" OR parasuicide*) OR KW("self-injur*" OR "self-harm" OR selfharm OR suicid* OR "self-kill*" OR "self-poison*" OR "self-stabb*" OR parasuicide*)

Psychotherapy & online interventions
DE "Multisystemic Therapy" OR DE "Play Therapy" OR DE "Psychotherapy" OR DE "Adlerian Psychotherapy" OR DE "Adolescent Psychotherapy" OR DE "Affirmative

© 2025 Janssen WC et al. *JAMA Network Open*.

Therapy" OR DE "Analytical Psychotherapy" OR DE "Autogenic Training" OR DE "Brief Psychotherapy" OR DE "Brief Relational Therapy" OR DE "Child Psychotherapy" OR DE "Client Centered Therapy" OR DE "Couples Therapy" OR DE "Eclectic Psychotherapy" OR DE "Emotion Focused Therapy" OR DE "Existential Therapy" OR DE "Experiential Psychotherapy" OR DE "Expressive Psychotherapy" OR DE "Feminist Therapy" OR DE "Gestalt Therapy" OR DE "Group Psychotherapy" OR DE "Guided Imagery" OR DE "Humanistic Psychotherapy" OR DE "Hypnotherapy" OR DE "Individual Psychotherapy" OR DE "Insight Therapy" OR DE "Integrative Psychotherapy" OR DE "Interpersonal Psychotherapy" OR DE "Logotherapy" OR DE "Narrative Therapy" OR DE "Network Therapy" OR DE "Persuasion Therapy" OR DE "Psychodrama" OR DE "Psychodynamic Psychotherapy" OR DE "Psychotherapeutic Counseling" OR DE "Rational Emotive Behavior Therapy" OR DE "Reality Therapy" OR DE "Solution Focused Therapy" OR DE "Strategic Therapy" OR DE "Supportive Psychotherapy" OR DE "Transactional Analysis" OR DE "Imaginal Exposure" OR DE "In Vivo Exposure" OR DE "Virtual Reality Exposure Therapy" OR DE "Conversion Therapy" OR DE "Exposure Therapy" OR DE "Implosive Therapy" OR DE "Systematic Desensitization Therapy" OR DE "Eye Movement Desensitization Therapy" OR DE "Cognitive Behavior Therapy" OR DE "Prolonged Exposure Therapy" OR DE "Behavior Therapy" OR DE "Cognitive Restructuring" OR DE "Cognitive Therapy" OR DE "Schema Therapy" OR DE "Mindfulness" OR DE "Acceptance and Commitment Therapy" OR DE "Dialectical Behavior Therapy" OR DE "Mindfulness-Based Interventions" OR DE "Psychotherapeutic Counseling" OR DE "Support Groups" OR DE "Counseling" OR DE "Group Counseling" OR DE "Computer Assisted Therapy" OR DE "Online Therapy" OR DE "Teleconsultation" OR DE "Telepsychiatry" OR DE "Telepsychology" OR DE "Telerehabilitation" OR DE "Teleconferencing" OR DE "Telemedicine" OR DE "Videoconferencing" OR TI(psychotherap* OR aromatherap* OR “art therap*” OR “behavioral therap*” OR “behavioral treatment*” OR “behavioral intervention*” OR “behavioural therap*” OR “behavioural treatment*” OR “behavioural intervention*” OR “behavior therap*” OR “behavior treatment*” OR “behavior intervention*” OR “behaviour therap*” OR “behaviour treatment*” OR “behaviour intervention*” OR CBT OR “cognitive psychotherap*” OR “cognitive therap*” OR bibliotherap* OR “color therap*” OR “crisis intervention*” OR “dance therap*” OR “emotion-focused therap*” OR “psychological feedback” OR “psychological treatment*” OR “psychological intervention*” OR “psychological therap*” OR “sensory feedback” OR “gestalt therap*” OR hypnos* OR imagery OR “implosive therap*” OR EMDR OR “psychological desensitization*” OR “desensitization reprocessing” OR “desensitisation reprocessing” OR “exposure therap*” OR “music therap*” OR grouptherap* OR “group therap*” OR “narrative therap*” OR mindfulness OR “play therap*” OR “psychoanalytic therap*” OR “reality therap*” OR “socioenvironmental therap*” OR “acceptance and commitment therap*” OR “milieu therap*” OR “problem-solving therap*” OR “dialectical behavio*” OR (DBT W3 dialectical*) OR “interpersonal therap*” OR (“future oriented” W3 training) OR “collaborative assessment*” OR “CAMS” OR (“systems training” W1 “emotional predictability” W1 “problem solving*”) OR “STEPPS” OR (“mentalisation-based” W3 training) OR “mentalisation-based therap*” OR (“mentalization-based” W3 training) OR “mentalization-based therap*” OR counselling OR counseling OR “self-help group*” OR “selfhelp group*” OR “motivational interview*” OR “blended face-to-face” OR telehealth OR “tele-health” OR telepsychology OR “tele-psychology” OR telepsychiatry OR “tele-psychiatry” OR “tele-

© 2025 Janssen WC et al. *JAMA Network Open*.

therap*” OR teletherap* OR “tele-medicine” OR telemedicine OR telecare OR “tele-care” OR teleconference* OR “tele-conferenc*” OR videoconferenc* OR “video-conferenc*” OR ehealth OR “e-health” OR “e-treat*” OR “e-therap*” OR mhealth OR “m-health” OR “internet-based*” OR “internet treat*” OR “internet intervention*” OR “internet counsel*” OR “distance counsel*” OR “web-based*” OR cybercounsel* OR “cyber-counsel*” OR “online treat*” OR “online therap*” OR “online intervention*” OR “online prevention*” OR “online counsel*” OR “e-counsel*” OR ecounsel* OR “serious game*” OR “serious gaming” OR gamification) OR AB(psychotherap* OR aromatherap* OR “art therap*” OR “behavioral therap*” OR “behavioral treatment*” OR “behavioral intervention*” OR “behavioural therap*” OR “behavioural treatment*” OR “behavioural intervention*” OR “behavior therap*” OR “behavior treatment*” OR “behavior intervention*” OR “behaviour therap*” OR “behaviour treatment*” OR “behaviour intervention*” OR CBT OR “cognitive psychotherap*” OR “cognitive therap*” OR bibliotherap* OR “color therap*” OR “crisis intervention*” OR “dance therap*” OR “emotion-focused therap*” OR “psychological feedback” OR “psychological treatment*” OR “psychological intervention*” OR “psychological therap*” OR “sensory feedback” OR “gestalt therap*” OR hypnos* OR imagery OR “implosive therap*” OR EMDR OR “psychological desensitization*” OR “desensitization reprocessing” OR “desensitisation reprocessing” OR “exposure therap*” OR “music therap*” OR grouptherap* OR “group therap*” OR “narrative therap*” OR mindfulness OR “play therap*” OR “psychoanalytic therap*” OR “reality therap*” OR “socioenvironmental therap*” OR “acceptance and commitment therap*” OR “milieu therap*” OR “problem-solving therap*” OR “dialectical behavio*” OR (DBT W3 dialectical*) OR “interpersonal therap*” OR (“future oriented” W3 training) OR “collaborative assessment*” OR “CAMS” OR (“systems training” W1 “emotional predictability” W1 “problem solving*”) OR “STEPPS” OR (“mentalisation-based” W3 training) OR “mentalisation-based therap*” OR (“mentalization-based” W3 training) OR “mentalization-based therap*” OR counselling OR counseling OR “self-help group*” OR “selfhelp group*” OR “motivational interview*” OR “blended face-to-face” OR telehealth OR “tele-health” OR telepsychology OR “tele-psychology” OR telepsychiatry OR “tele-psychiatry” OR “tele-therap*” OR teletherap* OR “tele-medicine” OR telemedicine OR telecare OR “tele-care” OR teleconference* OR “tele-conferenc*” OR videoconferenc* OR “video-conferenc*” OR ehealth OR “e-health” OR “e-treat*” OR “e-therap*” OR mhealth OR “m-health” OR “internet-based*” OR “internet treat*” OR “internet intervention*” OR “internet counsel*” OR “distance counsel*” OR “web-based*” OR cybercounsel* OR “cyber-counsel*” OR “online treat*” OR “online therap*” OR “online intervention*” OR “online prevention*” OR “online counsel*” OR “e-counsel*” OR ecounsel* OR “serious game*” OR “serious gaming” OR gamification) OR KW(psychotherap* OR aromatherap* OR “art therap*” OR “behavioral therap*” OR “behavioral treatment*” OR “behavioral intervention*” OR “behavioural therap*” OR “behavioural treatment*” OR “behavioural intervention*” OR “behavior therap*” OR “behavior treatment*” OR “behavior intervention*” OR “behaviour therap*” OR “behaviour treatment*” OR “behaviour intervention*” OR CBT OR “cognitive psychotherap*” OR “cognitive therap*” OR bibliotherap* OR “color therap*” OR “crisis intervention*” OR “dance therap*” OR “emotion-focused therap*” OR “psychological feedback” OR “psychological treatment*” OR “psychological intervention*” OR “psychological therap*” OR “sensory feedback” OR “gestalt therap*” OR hypnos* OR imagery OR “implosive therap*” OR

© 2025 Janssen WC et al. *JAMA Network Open*.

EMDR OR “psychological desensitization*” OR “desensitization reprocessing” OR “desensitisation reprocessing” OR “exposure therap*” OR “music therap*” OR grouptherap* OR “group therap*” OR “narrative therap*” OR mindfulness OR “play therap*” OR “psychoanalytic therap*” OR “reality therap*” OR “socioenvironmental therap*” OR “acceptance and commitment therap*” OR “milieu therap*” OR “problem-solving therap*” OR “dialectical behavio*” OR (DBT W3 dialectical*) OR “interpersonal therap*” OR (“future oriented” W3 training) OR “collaborative assessment*” OR “CAMS” OR (“systems training” W1 “emotional predictability” W1 “problem solving*”) OR “STEPPS” OR (“mentalisation-based” W3 training) OR “mentalisation-based therap*” OR (“mentalization-based” W3 training) OR “mentalization-based therap*” OR counselling OR counseling OR “self-help group*” OR “selfhelp group*” OR “motivational interview*” OR “blended face-to-face” OR telehealth OR “tele-health” OR telepsychology OR “tele-psychology” OR telepsychiatry OR “tele-psychiatry” OR “tele-therap*” OR teletherap* OR “tele-medicine” OR telemedicine OR telecare OR “tele-care” OR teleconference* OR “tele-conferenc*” OR videoconferenc* OR “video-conferenc*” OR ehealth OR “e-health” OR “e-treat*” OR “e-therap*” OR mhealth OR “m-health” OR “internet-based*” OR “internet treat*” OR “internet intervention*” OR “internet counsel*” OR “distance counsel*” OR “web-based*” OR cybercounsel* OR “cyber-counsel*” OR “online treat*” OR “online therap*” OR “online intervention*” OR “online prevention*” OR “online counsel*” OR “e-counsel*” OR ecounsel* OR “serious game*” OR “serious gaming” OR gamification)

RCT, CT & other studies
DE "Randomized Clinical Trials" OR DE "Random Sampling" OR DE "Clinical Trials" OR DE "Randomized Controlled Trials" OR TI(“Double-Blind Method” OR “Single-Blind Method” OR “Clinical trial*” OR "Randomized Controlled Trial*" OR “Random allocation” OR “Comparative stud*” OR “Evaluation stud*” OR “cross-over stud*” OR "pragmatic trial*" OR "real world trial*" OR ((singl* OR doubl* OR trebl* OR tripl*) W3 (mask* OR blind*)) OR "latin square" OR placebo* OR random* OR RCT OR control OR controll*) OR AB((“Double-Blind Method” OR “Single-Blind Method” OR “Clinical trial*” OR "Randomized Controlled Trial*" OR “Random allocation” OR “Comparative stud*” OR “Evaluation stud*” OR “cross-over stud*” OR "pragmatic trial*" OR "real world trial*" OR ((singl* OR doubl* OR trebl* OR tripl*) W3 (mask* OR blind*)) OR "latin square" OR placebo* OR random* OR RCT OR control OR controll*) OR KW(“Double-Blind Method” OR “Single-Blind Method” OR “Clinical trial*” OR "Randomized Controlled Trial*" OR “Random allocation” OR “Comparative stud*” OR “Evaluation stud*” OR “cross-over stud*” OR "pragmatic trial*" OR "real world trial*" OR ((singl* OR doubl* OR trebl* OR tripl*) W3 (mask* OR blind*)) OR "latin square" OR placebo* OR random* OR RCT OR control OR controll*)

Web of Science

# Suicide
TS=(“self-injur*” OR “self-harm” OR selfharm OR suicid* OR “self-kill*” OR “self-poison*” OR “self-stabb*” OR parasuicide*)

© 2025 Janssen WC et al. *JAMA Network Open*.

# Psychotherapy & online interventions
TS=(psychotherap* OR aromatherap* OR “art therap*” OR “behavioral therap*” OR “behavioral treatment*” OR “behavioral intervention*” OR “behavioural therap*” OR “behavioural treatment*” OR “behavioural intervention*” OR “behavior therap*” OR “behavior treatment*” OR “behavior intervention*” OR “behaviour therap*” OR “behaviour treatment*” OR “behaviour intervention*” OR CBT OR “cognitive psychotherap*” OR “cognitive therap*” OR bibliotherap* OR “color therap*” OR “crisis intervention*” OR “dance therap*” OR “emotion-focused therap*” OR “psychological feedback” OR “psychological treatment*” OR “psychological intervention*” OR “psychological therap*” OR “sensory feedback” OR “gestalt therap*” OR hypnos* OR imagery OR “implosive therap*” OR EMDR OR “psychological desensitization*” OR “desensitization reprocessing” OR “desensitisation reprocessing” OR “exposure therap*” OR “music therap*” OR grouptherap* OR “group therap*” OR “narrative therap*” OR mindfulness OR “play therap*” OR “psychoanalytic therap*” OR “reality therap*” OR “socioenvironmental therap*” OR “acceptance and commitment therap*” OR “milieu therap*” OR “problem-solving therap*” OR “dialectical behavio*” OR (DBT NEAR/3 dialectical*) OR “interpersonal therap*” OR (“future oriented” NEAR/3 training) OR “collaborative assessment*” OR “CAMS” OR (“systems training” NEAR/1 “emotional predictability” NEAR/1 “problem solving*”) OR “STEPPS” OR (“mentalisation-based” NEAR/3 training) OR “mentalisation-based therap*” OR (“mentalization-based” NEAR/3 training) OR “mentalization-based therap*” OR counselling OR counseling OR “self-help group*” OR “selfhelp group*” OR “motivational interview*” OR “blended face-to-face” OR telehealth OR “tele-health” OR telepsychology OR “tele-psychology” OR telepsychiatry OR “tele-psychiatry” OR “tele-therap*” OR teletherap* OR “tele-medicine” OR telemedicine OR telecare OR “tele-care” OR teleconference* OR “tele-conferenc*” OR videoconferenc* OR “video-conferenc*” OR ehealth OR “e-health” OR “e-treat*” OR “e-therap*” OR mhealth OR “m-health” OR “internet-based*” OR “internet treat*” OR “internet intervention*” OR “internet counsel*” OR “distance counsel*” OR “web-based*” OR cybercounsel* OR “cyber-counsel*” OR “online treat*” OR “online therap*” OR “online intervention*” OR “online prevention*” OR “online counsel*” OR “e-counsel*” OR ecounsel* OR “serious game*” OR “serious gaming” OR gamification)

# RCT, CT & other studies
TS=(“Double-Blind Method” OR “Single-Blind Method” OR “Clinical trial*” OR "Randomized Controlled Trial*" OR “Random allocation” OR “Comparative stud*” OR “Evaluation stud*” OR “cross-over stud*” OR "pragmatic trial*" OR "real world trial*" OR ((singl* OR doubl* OR trebl* OR tripl*) NEAR/3 (mask* OR blind*)) OR "latin square" OR placebo* OR random* OR RCT OR control OR controll*)

Scopus

Suicide
TITLE-ABS-KEY(“self-injur*” OR “self-harm” OR selfharm OR suicid* OR “self-kill*” OR “self-poison*” OR “self-stabb*” OR parasuicide*)

© 2025 Janssen WC et al. *JAMA Network Open*.

Psychotherapy & online interventions
TITLE-ABS-KEY(psychotherap* OR aromatherap* OR “art therap*” OR “behavioral therap*” OR “behavioral treatment*” OR “behavioral intervention*” OR “behavioural therap*” OR “behavioural treatment*” OR “behavioural intervention*” OR “behavior therap*” OR “behavior treatment*” OR “behavior intervention*” OR “behaviour therap*” OR “behaviour treatment*” OR “behaviour intervention*” OR CBT OR “cognitive psychotherap*” OR “cognitive therap*” OR bibliotherap* OR “color therap*” OR “crisis intervention*” OR “dance therap*” OR “emotion-focused therap*” OR “psychological feedback” OR “psychological treatment*” OR “psychological intervention*” OR “psychological therap*” OR “sensory feedback” OR “gestalt therap*” OR hypnos* OR imagery OR “implosive therap*” OR EMDR OR “psychological desensitization*” OR “desensitization reprocessing” OR “desensitisation reprocessing” OR “exposure therap*” OR “music therap*” OR grouptherap* OR “group therap*” OR “narrative therap*” OR mindfulness OR “play therap*” OR “psychoanalytic therap*” OR “reality therap*” OR “socioenvironmental therap*” OR “acceptance and commitment therap*” OR “milieu therap*” OR “problem-solving therap*” OR “dialectical behavio*” OR (DBT W/3 dialectical*) OR “interpersonal therap*” OR (“future oriented” W/3 training) OR “collaborative assessment*” OR “CAMS” OR (“systems training” W/2 “emotional predictability” W/2 “problem solving*”) OR “STEPPS” OR (“mentalisation-based” W/3 training) OR “mentalisation-based therap*” OR (“mentalization-based” W/3 training) OR “mentalization-based therap*” OR counselling OR counseling OR “self-help group*” OR “selfhelp group*” OR “motivational interview*” OR “blended face-to-face” OR telehealth OR “tele-health” OR telepsychology OR “tele-psychology” OR telepsychiatry OR “tele-psychiatry” OR “tele-therap*” OR teletherap* OR “tele-medicine” OR telemedicine OR telecare OR “tele-care” OR teleconference* OR “tele-conferenc*” OR videoconferenc* OR “video-conferenc*” OR ehealth OR “e-health” OR “e-treat*” OR “e-therap*” OR mhealth OR “m-health” OR “internet-based*” OR “internet treat*” OR “internet intervention*” OR “internet counsel*” OR “distance counsel*” OR “web-based*” OR cybercounsel* OR “cyber-counsel*” OR “online treat*” OR “online therap*” OR “online intervention*” OR “online prevention*” OR “online counsel*” OR “e-counsel*” OR ecounsel* OR “serious game*” OR “serious gaming” OR gamification)

RCT, CT & other studies
TITLE-ABS-KEY(“Double-Blind Method” OR “Single-Blind Method” OR “Clinical trial*” OR "Randomized Controlled Trial*" OR “Random allocation” OR “Comparative stud*” OR “Evaluation stud*” OR “cross-over stud*” OR "pragmatic trial*" OR "real world trial*" OR ((singl* OR doubl* OR trebl* OR tripl*) W/3 (mask* OR blind*)) OR "latin square" OR placebo* OR random* OR RCT OR control OR controll*)

Cochrane

Suicide
(“self-injur*” OR “self-harm” OR selfharm OR suicid* OR “self-killing” OR “self-poisoning” OR “self-stabbing” OR parasuicide*):ti,ab,kw

Psychotherapy & online interventions

© 2025 Janssen WC et al. *JAMA Network Open*.

(psychotherap* OR aromatherap* OR “art therapy” OR “behavioral therapy” OR “behavioral treatment” OR “behavioral intervention” OR “behavioural therapy” OR “behavioural treatment” OR “behavioural intervention” OR “behavior therapy” OR “behavior treatment” OR “behavior intervention” OR “behaviour therapy” OR “behaviour treatment” OR “behaviour intervention” OR CBT OR “cognitive psychotherapy” OR “cognitive therapy” OR bibliotherap* OR “color therapy” OR “crisis intervention” OR “dance therapy” OR “emotion-focused therapy” OR “psychological feedback” OR “psychological treatment” OR “psychological intervention” OR “psychological therapy” OR “sensory feedback” OR “gestalt therapy” OR hypnos* OR imagery OR “implosive therapy” OR EMDR OR “psychological desensitization” OR “desensitization reprocessing” OR “desensitisation reprocessing” OR “exposure therapy” OR “music therapy” OR grouptherap* OR “group therapy” OR “narrative therapy” OR mindfulness OR “play therapy” OR “psychoanalytic therapy” OR “reality therapy” OR “socioenvironmental therapy” OR “acceptance and commitment therapy” OR “milieu therapy” OR “problem-solving therapy” OR “dialectical behavior” OR (DBT NEAR/3 dialectical*) OR “interpersonal therapy” OR (“future oriented” NEAR/3 training) OR “collaborative assessment” OR “CAMS” OR (“systems training” NEAR/1 “emotional predictability” NEAR/1 “problem solving”) OR “STEPPS” OR (“mentalisation-based” NEAR/3 training) OR “mentalisation-based therapy” OR (“mentalization-based” NEAR/3 training) OR “mentalization-based therapy” OR counselling OR counseling OR “self help group” OR “selfhelp group” OR “motivational interviewing” OR “blended face-to-face” OR telehealth OR “tele health” OR telepsychology OR “tele psychology” OR telepsychiatry OR “tele psychiatry” OR “tele therapy” OR teletherap* OR “tele medicine” OR telemedicine OR telecare OR “tele care” OR teleconferenc* OR “tele conference” OR videoconferenc* OR “videoconferenc*” OR ehealth OR “e health” OR “e treatment” OR “e therapy” OR mhealth OR “m health” OR “internet based” OR “internet treatment” OR “internet intervention” OR “internet counselling” OR “distance counselling” OR “web based” OR cybercounsel* OR “cyber counselling” OR “online treatment” OR “online therapy” OR “online intervention” OR “online prevention” OR “online counselling” OR “e counselling” OR ecounsel* OR “serious games” OR “serious gaming” OR gamification):ti,ab,kw

© 2025 Janssen WC et al. *JAMA Network Open*.
