# Supplementary material for: Cognitive Behavior Therapy With and Without Narrative Assessment and Suicide Attempts: A Systematic Review and Meta-Analysis
Source: JAMA Netw Open. 2025 Nov 20;8(11):e2544632. doi: 10.1001/jamanetworkopen.2025.44632 (PMC12635872; doi:10.1001/jamanetworkopen.2025.44632)
Supplement: Supplement 2. — Data Sharing Statement [file jamanetwopen-e2544632-s002.pdf]

Data Sharing Statement

Janssen. Cognitive Behavior Therapy With and Without Narrative Assessment and Suicide Attempts. *JAMA Netw Open*. Published November 20, 2025.
doi:10.1001/jamanetworkopen.2025.44632

Data
Data available: No

Additional Information
Explanation for why data not available: it is already publicly available through the public dataset we used (MetaPsy)
